# Adaptation and evolution of deep-sea scale worms (Annelida: Polynoidae): insights from transcriptome comparison with a shallow-water species

**DOI:** 10.1038/srep46205

**Published:** 2017-04-11

**Authors:** Yanjie Zhang, Jin Sun, Chong Chen, Hiromi K. Watanabe, Dong Feng, Yu Zhang, Jill M.Y. Chiu, Pei-Yuan Qian, Jian-Wen Qiu

**Affiliations:** 1 Department of Biology, Hong Kong Baptist University, Hong Kong, P. R. China; 2 Division of Life Sciences, The Hong Kong University of Science and Technology, Clear Water Bay, Hong Kong, P. R. China; 3 Department of Subsurface Geobiological Analysis and Research, Japan Agency for Marine-Earth Science and Technology, 2-15 Natsushima-cho, Yokosuka, Kanagawa, 237-0061, Japan; 4 Department of Marine Biodiversity Research, Japan Agency for Marine-Earth Science and Technology, 2-15 Natsushima-cho, Yokosuka, Kanagawa, 237-0061, Japan; 5 CAS Key Laboratory of Marginal Sea Geology, South China Sea Institute of Oceanology, Chinese Academy of Sciences, Guangzhou, 510301, P. R. China; 6 College of Life Sciences and Oceanography, Shenzhen University, Shenzhen, P. R. China

## Abstract

Polynoid scale worms (Polynoidae, Annelida) invaded deep-sea chemosynthesis-based ecosystems approximately 60 million years ago, but little is known about their genetic adaptation to the extreme deep-sea environment. In this study, we reported the first two transcriptomes of deep-sea polynoids (*Branchipolynoe pettiboneae, Lepidonotopodium* sp.) and compared them with the transcriptome of a shallow-water polynoid (*Harmothoe imbricata*). We determined codon and amino acid usage, positive selected genes, highly expressed genes and putative duplicated genes. Transcriptome assembly produced 98,806 to 225,709 contigs in the three species. There were more positively charged amino acids (i.e., histidine and arginine) and less negatively charged amino acids (i.e., aspartic acid and glutamic acid) in the deep-sea species. There were 120 genes showing clear evidence of positive selection. Among the 10% most highly expressed genes, there were more hemoglobin genes with high expression levels in both deep-sea species. The duplicated genes related to DNA recombination and metabolism, and gene expression were only enriched in deep-sea species. Deep-sea scale worms adopted two strategies of adaptation to hypoxia in the chemosynthesis-based habitats (i.e., rapid evolution of tetra-domain hemoglobin in *Branchipolynoe* or high expression of single-domain hemoglobin in *Lepidonotopodium* sp.).

The discoveries of hydrothermal vents in the late 1970s and cold seeps in the early 1980s have revolutionized our view of life in the deep sea. Hydrothermal vents discharge geothermally heated water rich in methane, hydrogen sulfide and other hydrocarbon-rich fluids, while cold seeps release the same dissolved organic chemicals slowly without causing an appreciable temperature rise^1^. Despite the differences in the source of reduced organic compounds and temperature in the fluids, hydrothermal vents and cold seeps are both productive ecosystems in the deep sea^2^, and they can share more than 20% species when occur in the same region, indicating their genetic connectivity^3^.

There has been great interest in understanding how animals have adapted to the extreme conditions in these habitats that are characterized by high pressure, darkness, variable temperatures and high concentrations of toxic substances^4^,^5^,^6^,^7^,^8^,^9^. However, except for few species^10^,^11^, the limited genomic resources have hindered studies of physiological adaptation at the molecular level for animals living in these extreme environments.

Advances in sequencing technologies over the last few years have provided unprecedented opportunities for biologists. To date, these technologies have been applied in various areas of genomic research including whole genome sequencing, whole transcriptome sequencing, targeted resequencing, and noncoding RNA sequencing. Among these research areas, transcriptome sequencing, which targets the expressed gene transcripts, has been applied widely to gene discovery and to understanding the genetic base of adaptation to extreme environments due to its high affordability^12^,^13^. For instance, transcriptome sequencing was applied to analyze gene duplication in an Antarctic icefish, and found that the duplicated genes are highly enriched in mitochondrial functions^14^. It was used to understand the adaptation of a highland fish, which showed that genes related to hypoxia and energy metabolism have undergone rapid evolution^15^. This technique was also applied to analyze several depth-related genes (LDH-A, LDH-B, MDHc, ACTA1 isoform 1) in a deep-sea fish which indicated that these genes are constrained by purifying selection^16^. Moreover, transcriptome analysis of a hydrothermal vent annelid showed that high frequency of charged versus polar residues can be responsible for its thermo-adaptation^17^.

Scale worms in the family Polynoidae are well represented from the shallow intertidal to the deep sea^18^,^19^,^20^. In the deep sea, more than 120 species in 53 genera of polynoids have been reported from various chemosynthesis-based habitats, including hydrothermal vents, cold seeps, sunken wood and whale carcasses^21^,^22^. These scale worms are believed to be the descendants of shallow-water species that invaded the deep sea ca. 60 million years ago, and thereafter diverged to colonize various chemosynthesis-based habitats across the world’s oceans^23^. Members of the genus *Branchipolynoe* (Polynoidae) are commonly found in the mantle cavity of *Bathymodiolus* mussels living in hydrothermal vents and cold seeps^24^,^25^. Currently this genus has three species described from different biogeographic areas^26^: *B. symmytilida* from the hydrothermal vents of the Galápagos Rift in the eastern Pacific^27^, *B. seepensis* from the cold-seeps of the Florida Escarpment in the Gulf of Mexico^28^, and *B. pettiboneae* from the hydrothermal vents of the Okinawa Trough and the Kaikata Seamount in the northwestern Pacific^29^. Undescribed species of *Branchipolynoe* have been reported in other regions, such as the Central Indian Ridge^1^ and sunken vegetation off Papua New Guinea^30^. The widespread geographic occurrence of *Branchipolynoe* spp. makes them an excellent model in studies of population genetics, speciation, and biogeography^31^,^32^. Moreover, previous studies have shown that *Branchipolynoe* spp. have some morphological and molecular adaptations that have allowed them to thrive in the deep-sea chemosynthesis habitats that often experience hypoxia^33^,^34^, making them an excellent model for studies of adaptive evolution. Specifically, their parapodia bear well-developed arborescent branchiae, which are used to facilitate gaseous exchange with the environment^35^. Their coelomic fluid is red, containing high concentrations of hemoglobin for oxygen storage and transport. These morphological and molecular adaptations are believed to be essential for their survival in the chemosynthesis habitats that are often hypoxic, with oxygen concentrations being less than 2 mg/L^1^,^36^,^37^. The hemoglobins in *B. symmytilida* and *B. seepensis* have high affinity to oxygen; it has a tetra-domain structure that is unique among the hemoglobins of polychaetes^23^,^34^. *Lepidonotopodium* spp. are free-living polynoids in deep-sea hydrothermal vents^21^. They do not bear branchiae but their coelomic fluid is also red and contains a one-domain hemoglobin^23^.

To gain insight into the genetic basis of adaptation of deep-sea polynoids to extreme environmental conditions, we sequenced the transcriptomes of *B. pettiboneae* and *Lepidonotopodium* sp., and compared them with that of the shallow-water polynoid *Harmothoe imbricata*, which is in a clade of polynoids that is closest in phylogenetic relationship with all deep-sea polynoids^20^. *Harmothoe imbricata* is commonly found in low intertidal and subtidal rocky shores of North America and northern Europe^37^. We aim to provide genomic resources for the deep-sea polynoids, and discover several aspects of their transcriptomic differences with the shallow-water species, including codon and amino acid usage, expression of highly expressed genes, composition of duplicated genes, as well as evolutionary patterns of selected genes that may have undergone positive selection. In a broader sense, the findings will help us better understand how animals have adapted to deep-sea extreme environments.

## Materials and Methods

### Obtaining high-throughput cDNA sequences

Specimens of *Branhipolynoe pettiboneae* were collected from Jiaolong Ridge, a cold seep in the South China Sea (22°06.921′N, 119°17.131′E, depth 1122 m) in June 2013 during the first scientific cruise of the manned submersible *Jiaolong*. At this site, *B. pettiboneae* lived inside the mantle cavity of the deep-sea mussel *Bathymodiolus platifons*. The site temperature was 3.6 °C, but dissolved oxygen concentration was not measured. Specimens of *Lepidonotopodium* sp. were collected from the Noho site, a hydrothermal vent in the Okinawa Trough (27°31.0′N, 126°58.9′E, depth 1550 m), using ROV *Kaiko MK-IV* on-board R/V *Kairei* during the cruise KR15-17 in November 2015. The site temperature was 3.84 °C, and dissolved oxygen concentration was 1.76 mg/L.

The samples were kept in partially insulated sample boxes, and were preserved in RNALater (Ambion, Austin, TX) immediately once they were brought to the deck, which was roughly three hours after sampling. The samples were later transported to Hong Kong Baptist University on dry ice. For both deep-sea species, total RNA was extracted from three middle parapodia with elytra of a specimen using TRIzol reagent (Invitrogen, Carsbad, NM, USA) following the manufacturer’s protocol. The quality of RNA samples were detected by 1% agarose gel electrophoresis and stained using GelRed (Biotium, Fremont, CA, USA), then visualized under ultraviolet light. The quantity of RNA samples were analyzed using Bioanalyzer 2100 (Agilent Technologies, CA, USA). The RNA samples were sent to Beijing Genomics Institute (BGI), Shenzhen for messenger RNA enrichment, RNA fragmentation, cDNA synthesis, adapter ligation, and PCR amplification^38^, and the PCR product was sequenced to produce paired-end reads of 100 bp read length for *B. pettiboneae* using an Illumina Hiseq 2000 in 2014, and 150 bp read length for *Lepidonotopodium* sp. using an Illumina Hiseq 4000 in 2015. The raw transcriptomic data for *Branchipolynoe pettiboneae* and *Lepidonotopodium* sp. are deposited in NCBI SRA database with accession number of SRR4419842 and SRR4419843, respectively. Raw transcriptome sequences of *Harmothoe imbricata* were obtained from NCBI’s SRA database^39^ (accession no. SRX1015591). The RNA of this sample was extracted from an elytra of a middle parapodia collected from the intertidal zone of a rocky shore at Land’s End (43.807 °N, 69.995 °W) on Bailey Island, Maine, USA^39^. Below the rocky shore is a sandy beach and the seawater at the site is well-oxygenated.

### Transcriptome assembly and annotation

The raw reads were filtered using Trimmomatic v0.33^40^. In brief, adapters were removed based on information provided in the TruSeq RNA Library Prep Kit which was used in RNA library preparation (*B. pettiboneae* and *H. imbricata*: ILLUMINACLIP:TruSeq2-PE.fa:2:30:10; *Lepidonotopodium* sp.: ILLUMINACLIP:TruSeq3-PE.fa:2:30:10), the leading and trailing low quality bases were removed (LEADING:3 TRAILING:3), and cut when the average quality per base was below 15 with a 4-base wide sliding window, and removed when the length was below 36 bases (SLIDINGWINDOW:4:15 MINLEN:36). Trimmed paired-end reads of the three species were used for assembly using Trinity trinityrnaseq-2.0.6^41^. RSEM 1.2.19^42^ was used to estimate the transcript abundance, and the lowly expressed transcript isoforms (IsoPct < 1%) were removed. TransDecoder was then used to identify the candidate open reading frames (ORFs) and peptides in the filtered transcripts. Identical sequences (100% similarity) in predicted peptides were removed by CD-HIT^43^. The longest predicted protein of each contig was selected using a python script (Supplementary Information, PickUpLongest.py). Annotation was carried out by InterProScan-5.13-52.0^44^. A Gene Ontology (GO) annotation plot was made using WEGO^45^.

### Calculation of GC content and codon usage

The overall GC content and codon usage of transcriptome from *B. pettiboneae, Lepidonotopodium* sp. and *H. imbricata* were calculated using codonW 1.4.4^46^. Relative synonymous codon usage (RSCU) was used to indicate the codon usage pattern. RSCU = 1 indicates that the codons are used equally and randomly. RSCU > 1 and RSCU < 1 indicates positive and negative codon usage bias, respectively. Codon usage bias was considered strong when absolute ΔRSCU > 0.1^47^. The proportion of each amino acid is the summation of all its codons.

### Identification of orthologous genes and Ka/Ks analysis

Orthologs were identified by pair-wise comparison using OrthoMCL^48^. The predicted protein sequences with high identity ( > 95%) were removed with CD-HIT to eliminate isoforms caused by alternative splicing. Default settings were used for the OrthoMCL analysis and aligned sequences with less than 50 amino acids were excluded. Only 1:1 orthologous genes were used for base substitution analysis. The input files for Ka/Ks_Calculator^49^ were prepared using ParaAT1.0^50^. A maximum likelihood method with the MLPB model was used to calculate non-synonymous substitutions per nonsynonymous site (*Ka*), synonymous substitutions per synonymous site (*Ks*), and the ratio between *Ka* and *Ks* (Ka/Ks). Only ortholog pairs with *P*-value (Fisher’s exact test) <0.05, 0.01 < Ks < 1, and Ka < 1 were retained^51^. Ka/Ka > 1 was considered as a clear signal of positive selection. Positively selected genes were annotated based on the GO annotation and InterPro online database^52^.

### Comparison of highly expressed genes in three species

Gene expression level was estimated using RSEM 1.2.19. Based on the reads per kilobase of exon per million reads mapped (FPKM), only highly expressed annotated genes (top 10%) were included in the comparison. The 10% highly expressed genes were classified based on their functional annotation. Their percentage and average expression level were compared to illustrate the differences in cellular processes among the three species.

### Identification of putatively duplicated genes

Putatively duplicated genes were identified using OrthoMCL based on an *E* value of 1e × 10^−5^ in BLASTP^53^. Alignments shorter than 50 amino acid residues (i.e. <150 nt) were removed. Pairs of peptide sequences with similarity between 70% and 98% were considered as duplicated genes^14^. Only clusters with annotation were included in the comparison. Gene Ontology classification was made using WEGO. Functional enrichment analysis was done using GOEAST^54^. Specifically, the Benjamini and Yekutieli false discovery rate correction under dependency was applied. GO terms with *p* < 0.05 were considered as significantly enriched based on a hypergeometric statistical test.

### Phylogenetic and evolutionary analyses of hemoglobins

To illustrate the use of the transcriptome data, we conducted phylogenetic and evolutionary analyses of hemoglobins in deep-sea scale worms. This group of proteins function in oxygen binding, and could be important in the adaptation of deep-sea scale worms to the hypoxic conditions in chemosynthesis-based ecosystems^5^,^23^. Potential hemoglobin sequences were fetched from the assembled transcriptome of the three scale worms using BLASTP with an *E* value of 1e × 10^−5^. Hemoglobin sequences from other deep-sea scale worms were also obtained from GenBank. Phylogenetic analyses were conducted using the Maximum Parsimony (MP) and the Maximum Likelihood (ML) methods. A total of 18 hemoglobin sequences from the other deep-sea scale worms including *B. seepensis* and *B. symmytilida* obtained from GenBank were added in the alignment and phylogenetic analysis. Several intercellular hemoglobin sequences from *Glycera dibranchiata* (family Glyceridae), and extracellular hemoglobin sequences from *Lumbricus terrestris* (family Lumbricidae, Oligochaeta) were used as outgroups. Accession numbers for the hemoglobin sequences are listed in Supplementary Table S1. All the sequences were aligned using Muscle3.8.31 implemented in Mesquite^55^. The MP tree was constructed using PAUP* V4.0^56^. The ML analysis was conducted using RaXML GUI1.3^57^ with the Dayhoff +I+G model which was determined using ProtTest-3.0^58^. The bootstrap values were all based on 1000 iterations. The candidate single domain and tetra-domain sequences of *B. pettiboneae* were confirmed based on their similarities with characterized hemoglobins obtained from the phylogenetic analysis. The hemoglobin sequences were analyzed for positive selection using PAML 4.8 following a previous study^34^.

## Results

### Transcriptome assembly and annotation

**Figure 1:**
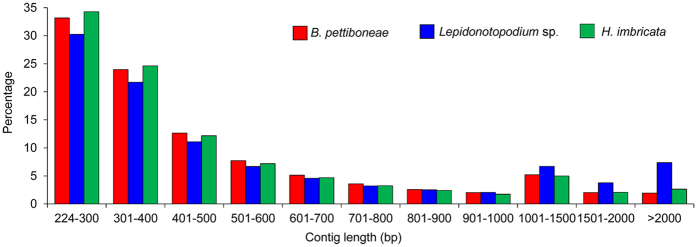
Contig length distribution in the assembled transcriptomes of *B. pettiboneae, Lepidonotopodium* sp. and *H. imbricata*.

**Figure 2:**
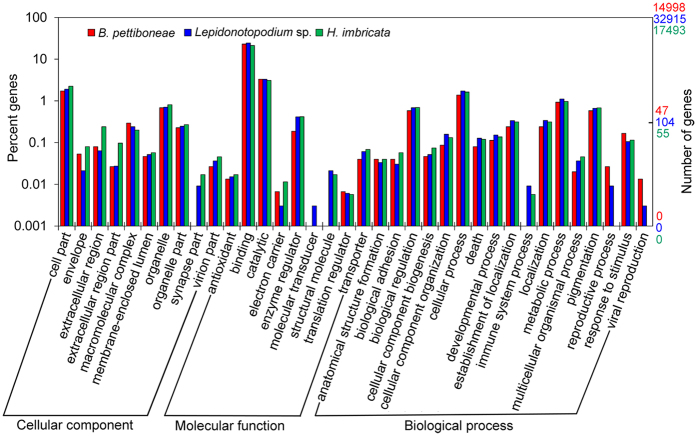
Gene Ontology (GO) distribution of annotated genes in the transcriptomes of *B. pettiboneae, Lepidonotopodium* sp. and *H. imbricata*. Genes are grouped into three main functional categories: biological process, cellular component and molecular function. The left abscissa indicates the percentage and right one shows the number of annotated genes. One gene may have match in multiple GO terms.

There are 3.8 Gb raw reads in *B. pettiboneae*, 7.0 Gb raw reads in *Lepidonotopodium* sp., and 3.9 Gb raw reads in *Harmothoe imbricata* (Table 1). Removing adaptors and low-quality reads resulted in the retention of 98.4%, 84.7% and 91.3% reads for the three species, respectively. The cleaned reads were used for the assembly and generated 153,339 contigs for *B. pettiboneae*, 225,709 contigs for *Lepidonotopodium* sp. and 98,806 contigs for *H. imbricata*. The assembled transcriptome had an average contig length of 521 to 738 bp with N50 of 583 to 1,198 in the three species. Contig length distributions for the three species are shown in Fig. 1. Short contigs (<1,000 bp) constituted about one third in the three species, but there were about 8% more long contigs ( > 1,000) in *Lepidonotopodium* sp. There were 38,734, 79,930 and 36,001 predicted proteins in *B. pettiboneae, Lepidonotopodium* sp. and *H. imbricata*, respectively. Applying InterProScan revealed 38.7%, 41.2% and 48.6% annotated genes for *B. pettiboneae, Lepidonotopodium* sp. and *H. imbricata*, respectively (Table 1; Supplementary Table S2). A WEGO gene ontology annotation plot (Fig. 2) showed that the compositions of genes in the three species were broadly similar in cellular components, molecular functions and biological processes.

### Comparison of GC content and codon usage

The GC content of the *B. pettiboneae, Lepidonotopodium* sp. and *H. imbricata* transcriptome is 52.1%, 53.2% and 52%, respectively. The difference in codon usage ranges from 0 to 5.14‰, and the difference in Relative Synonymous Codon Usage (RSCU) ranges from 0 to 0.24. Most of the codons have very similar proportions and RSCU values among the three species. However, 14 codons have strong negative or positive codon usage bias (absolute ΔRSCU > 0.1) in the deep-sea species compared with the shallow-water species (Supplementary Table S3). More than half of the codons with strong biased ΔRSCU values encode the amino acids glycine, proline and cysteine. The usage pattern of amino acids is listed in Table 2. The species difference in proportion for more than half of the amino acids is less than 0.2%. However, there are large differences ( > 0.4%) for several amino acids between the deep-sea and shallow-water species. Specifically, there are more positively charged amino acids (i.e., histidine and arginine) and less negatively charged amino acids (i.e., aspartic acid and glutamic acid) in the deep-sea species. In addition, there are more non-polar amino acid leucine and less glycine in the deep-sea species.

### Positively selected genes

**Figure 3:**
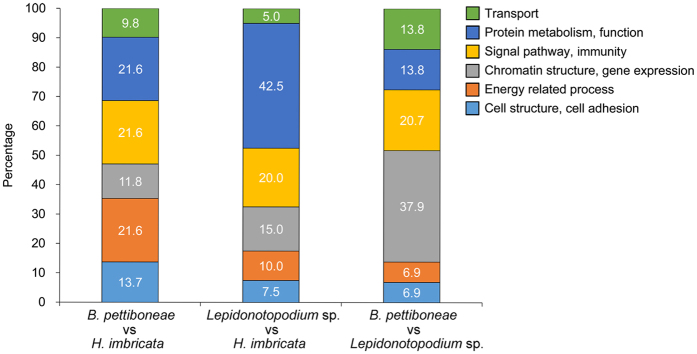
Biological functions of positively selected genes in pair-wise comparisons among the three species of scale worms.

There were 5,494, 6,851 and 7,311 pairs of orthologs between *B. pettiboneae* and *H. imbricata, Lepidonotopodium* sp. and *H. imbricata*, and *B. pettiboneae* and *Lepidonotopodium* sp., respectively (Table 3). Filtering reduced the numbers to 1,161, 1,801 and 3,269 for the three pairs, respectively. Base substitution analysis revealed a clear signal of positive selection (Ka/Ks > 1) in 192 orthologs between *B. pettiboneae* and *H. imbricata*, 306 orthologs between *Lepidonotopodium* sp. and *H. imbricata*, and 416 orthologs between *B. pettiboneae* and *Lepidonotopodium* sp. However, only 51, 40 and 29 positive selected genes were annotated in the three pair-wise comparisons, respectively (Table 3 and Supplementary Table S4). These positively selected genes are mainly related to six different functions: cell structure and cell adhesion, energy-related process, chromatin structure and gene expression, signal pathway and immunity, protein metabolism and function, and transport (Fig. 3). Among the positively selective orthologs between *B. pettiboneae* and *H. imbricata*, genes involved in protein metabolism, signal pathway, immune, and energy-related process accounted for the largest proportions (64% in total). Between *Lepidonotopodium* sp. and *H. imbricata*, the largest proportion of positively selected genes is related to protein metabolism and function (43%), followed by signal pathway and immunity (20.0%). Between the two deep-sea species, more than one third of positively selected genes (38%) are involved in chromatin structure and gene expression, and one fifth are involved in signal pathway and immunity.

### Comparison of highly expressed genes

**Figure 4:**
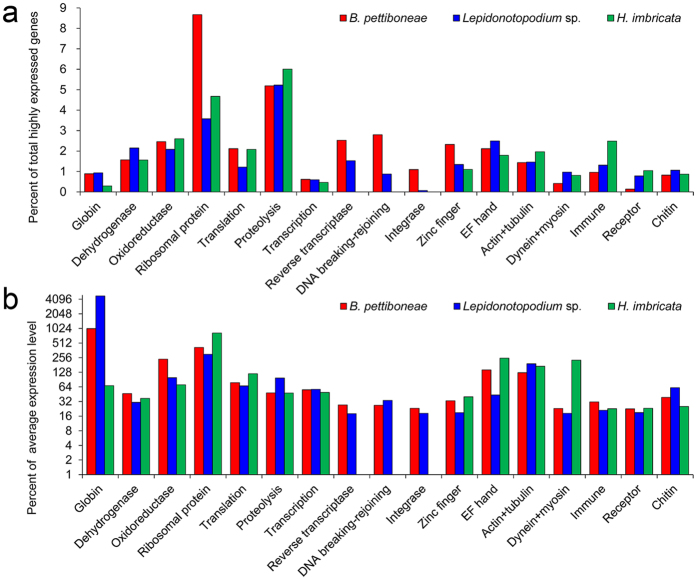
Comparison of highly expressed genes in *B. pettiboneae, Lepidonotopodium* sp. and *H. imbricata*. (**a**) Percentage of genes participated in different cellular processes. Each datum shows the percentage each gene group to all top 10% highly expressed genes. (**b**) Expression level for gene groups participated in different cellular processes. Each datum shows the percentage of average FPKM value of a gene group to average FPKM value of all top 10% highly expressed genes.

The top 10% highly expressed genes included 1465 genes in *B. pettiboneae*, 3215 genes in *Lepidonotopodium* sp. and 1733 genes in *H. imbricata*. These genes had FPKM value ranging from 7.78 to 4527.82 (average 72.8), from 8.77 to 60511.8 (average 117.9) and from 10.22 to 2897.81 (average 80.7) in *B. pettiboneae, Lepidonotopodium* sp. and *H. imbricata*, respectively (Supplementary Table S5). Gene groups related to conserved biological processes are compared with respect to their percentage (Fig. 4a) and expression level (Fig. 4b). Gene groups related to several biological processes, including dehydrogenase, transcription, translation, proteolysis and cytoskeleton (actin and tubulin), were of comparable expression levels in the three species. Nevertheless, there were notable differences in the expression of several gene groups among the three species. In particular, globin genes in both deep-sea species were more abundant (about three times) and had higher expression levels (more than ten times) than in the shallow-water species. Gene groups involved in DNA recombination and integration including reverse transcriptase, DNA breaking-rejoining domains and integrase were only present and highly expressed in the two deep-sea species (Fig. 4). Zinc finger and EF hand, which are common protein-DNA binding domains, were more abundant in the two deep-sea species (Fig. 4a).

### Comparison of putatively duplicated genes

**Figure 5:**
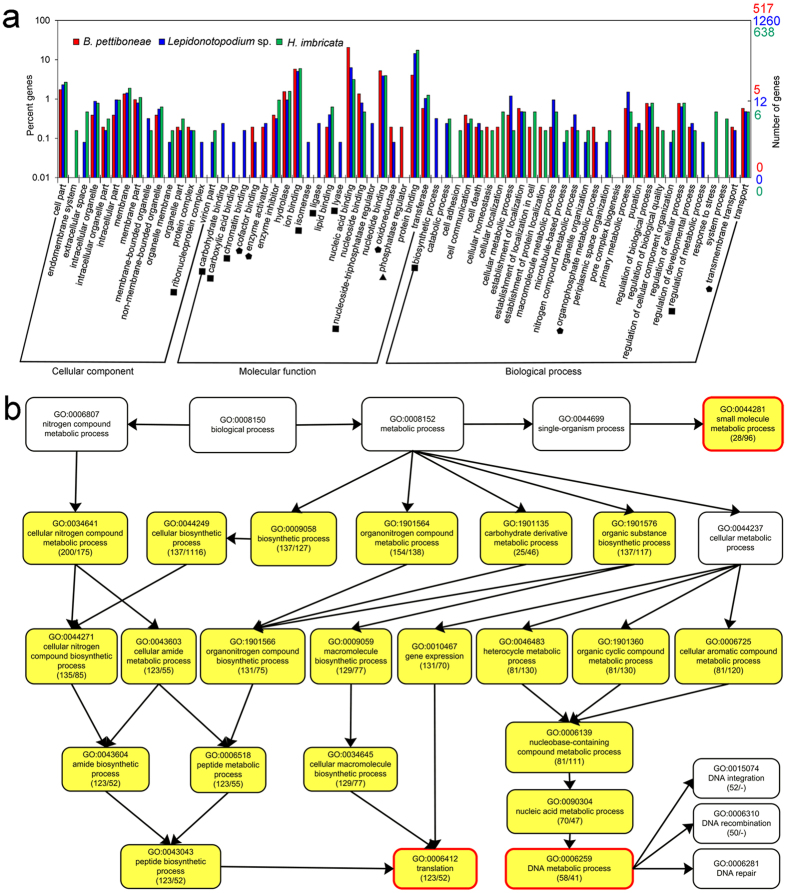
Comparison of putatively duplicated genes in three species of scale worms. (**a**) Gene Ontology (GO) distribution of putatively duplicated genes in the three species. The triangles stand for groups only present in *B. pettiboneae*; the squares for groups only present in *Lepidonotopodium* sp.; the pentagons for groups present in both deep-sea species. (**b**) Significantly enriched biological process GO terms of putatively duplicated genes only present in both deep-sea species (highlighted in yellow). Boxes framed in red show terminal GO terms in both deep-sea species. Data in parentheses include the number of genes associated with the listed GO term in *B. pettiboneae* (first number) and *Lepidonotopodium* sp. (second number). A dash in parenthesis indicates absence of the gene. Arrows represent the connection between two enriched GO terms at different levels. Only selected significantly enriched GO branches in deep-sea species are shown to illustrate the interspecific differences with shallow-water species.

There were 517, 1260 and 638 annotated duplicated gene clusters in *B. pettiboneae, Lepidonotopodium* sp. and *H. imbricata*, respectively. Their average duplicated copies were 3.6, 3.0 and 2.5 in the three species, respectively (Supplementary Table S6). GO functional annotation results of the duplicated clusters are shown in Fig. 5a. Compared with the shallow-water species, only the two deep-sea species have duplicated genes involved in cofactor binding, enzyme inhibitor and oxidoreductase activity of molecular function, and transmembrane transport of biological process. Several GO terms were only present in one deep-sea species, such as phosphatase regulator in *B. pettiboneae*; ribonucleoprotein complex of cellular component, carbohydrate binding, carboxylic acid binding, chromatin binding, isomerase, ligase, nucleside-triphosphatase regulator, biosynthetic process and regulation of metabolic process in *Lepidonotopodium* sp. The results of functional enrichment are shown in Supplementary Table S7. There were 57 GO terms significantly enriched for putatively duplicated genes in *B. pettiboneae*, 116 in *Lepidonotopodium* sp., and 37 in *H. imbricata*. There were 23 GO terms enriched only in the two deep-sea species (Fig. 5b), including three terminal GO terms: small molecular metabolic process, translation and DNA metabolic process. The first is related to carbohydrate and protein metabolism including pyruvate kinase and citrate synthase; and aminoacyl-tRNA synthetase and Peptidase are involved in protein metabolism. Genes enriched in translation are mainly ribosomal proteins that are responsible for protein synthesis. Genes enriched in DNA metabolism are involved in DNA recombination, integration and DNA repair.

### Hemoglobins

**Figure 6:**
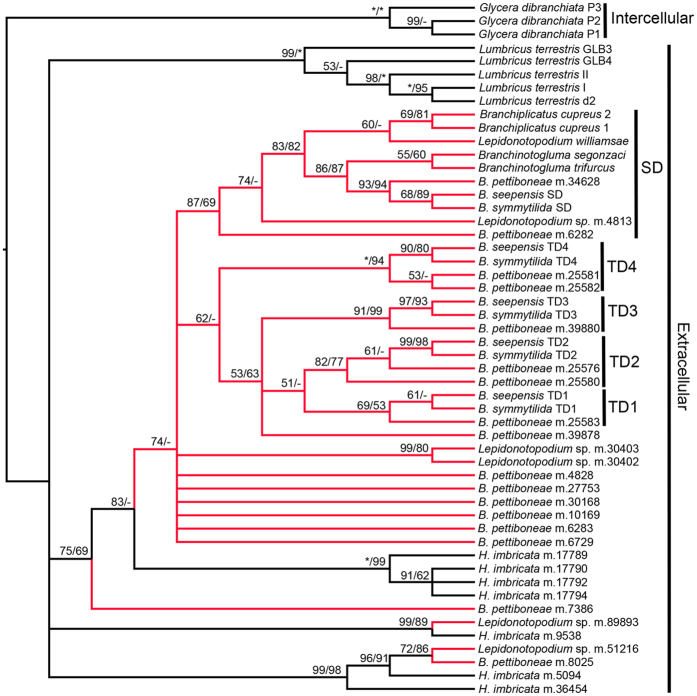
Phylogenetic tree of potential hemoglobin sequences from scale worms. Sequences from *Lumbricus* and *Glycera* served as outgroups. Numbers above branches represent MP/ML bootstrap values based on 1,000 iterations, with 100 (indicated using an asterisk) as the highest value. Bootstrap values below 50 are shown only as a short dash due to the weak support. Sequences from deep-sea species are highlighted in red color.

There were 17 extracellular hemoglobin sequences from *B. pettiboneae*, 5 from *Lepidonotopodium* sp. and 7 from *H. imbricata*. Phylogenetic analysis showed that most of the hemoglobin sequences from the deep-sea scale worms are nested in a major clade (highlighted in red in Fig. 6). This result is consistent with the phylogenetic relationship reconstructed with concatenated sequences from the COI and 16S, 18S, and 28S rRNA genes that showed all deep-sea polynoids with a sequence in the NCBI database were derived from the same shallow-water common ancestor (Supplementary Fig. S1). There are 2 sequences from *Lepidonotopodium* sp. and 6 sequences from *B. pettiboneae* whose structures are unknown. Ten single-domain hemoglobins from 2 species of *Lepidonotopodium* sp., 1 species of *Branchiplicatus*, 2 species of *Branchinologluma*, and 3 species of *Branchipolynoe* are clustered in a clade, which is sister to a clade including all the tetra-domain hemoglobins from three species of *Branchipolynoe* including *B. pettiboneae, B. seepensis* and *B. symmytilida*. In each of the four domains, the *B. pettiboneae* sequence is sister to a clade containing the *B. seepensis* and *B. symmytilida* sequences, indicating the tetra-domain hemoglobins were present before the divergence of the *Branchipolynoe* spp. and the level of divergence is related to the geographic distribution of these species. These species with tetra-domain hemoglobin are in a terminal branch of the phylogenetic tree, with *Branchinotogluma* (a species with single-domain hemoglobin) as the sister taxon, and this result is also consistent with the phylogenetic tree based on the concatenated COI and 16S, 18S, and 28S rRNA sequences (Supplementary Fig. S1).

**Figure 7:**
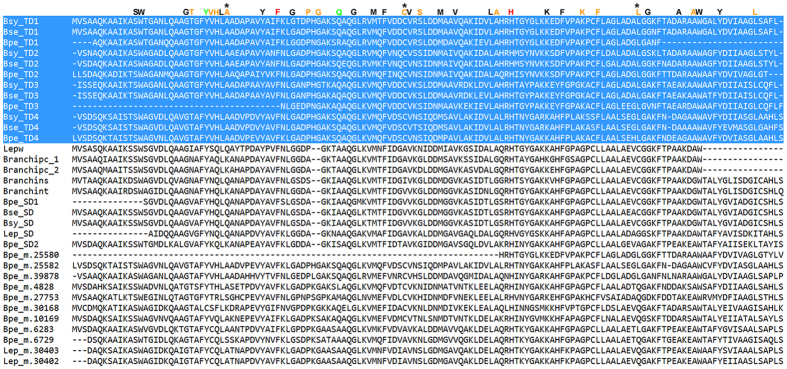
Alignment of hemoglobin sequences from deep-sea polynoids. The sequences with a blue background are tetra-domain hemoglobins from three species of *Branchipolynoe*. Green letters above sequences indicate the positions of the heme pocket residues in tetra-domain hemoglobin. Red letters indicate the positions of conserved amino acid residues in all hemoglobin domains. Black letters show the positions of conserved amino acids in all hemoglobins from deep-sea polynoids. Orange letters show the positions of conserved amino acids in tetra-domain hemoglobins. The asterisks show positive selected sites in tetra-domain hemoglobins. SD: single-domain; TD: tetra-domain; Bse: *B. seepensis*; Bsy: *B. symmytilida*; Bpe: *B. pettiboneae*; Lepw: *Lepidonotopodium williamsae*; Branchipc: *Branchiplicatus cupreus*; Branchins: *Branchinotogluma segonzaci*; Branchint: *Branchinotogluma trifurcus*.

Alignment of the single- and tetra-domain hemoglobin sequences showed that more than a quarter of amino acids (39/140) are conserved, and the first half of the sequences is more conserved than the second half (Fig. 7). Heme pocket residues, which explain the high oxygen affinity^23^, are conserved in tetra-domain hemoglobin sequences. There are 15 conserved amino acids in the tetra-domain sequences. Base substitution analyses of single-domain and tetra-domain hemoglobins in *Branchipolynoe* spp. using the branch-site model in PAML showed that three of the tetra-domains (except TD1) carry a clear signal of positive selection (Table 4). There are three sites having undergone positive selection in the tetra-domain hemoglobins (Table 4, Fig. 7).

## Discussion

We provided transcriptome resources for the deep-sea scale worms *Branchipolynoe pettiboneae* and *Lepidonotopodium* sp. by high-throughput next-generation sequencing and *de novo* assembly. These are the only transcriptome resources for this group of scale-bearing polychaetes that are widely distributed in deep-sea chemosynthesis-based ecosystems. The resources can be used to support physiological, phylogenetic and evolutionary studies at the molecular level.

Our analysis of the codon usage and amino acid composition showed that there were differences in these two parameters between the deep-sea and shallow-water species, which is consistent with the case between deep-sea and intertidal nematodes^9^. In addition, there are more basic polar amino acids (arginine and histidine), and less acidic polar amino acid (aspartic acid) in deep-sea polynoids, which is consistent with a previously study showing that basic polar amino acids are more abundant in organisms living in low temperatures than in high temperatures^59^. The change in amino acid composition has been considered an adaptation to low temperature, which could affect protein function^59^.

Changes in DNA or protein sequences can be adaptive because just a few mutations can significantly affect molecular function. For example, only two amino acid substitutions in key functional positions of the skeletal actin of deep-sea fish can reduce the volume change associated with actin polymerization, which maintains the DNase I activity of actin at high pressure^60^. However, it is important to distinguish nonsynonymous mutation that alters amino acid from synonymous mutation that does not change amino acid. Analyzing the transcriptome-wide rates of nonsynonymous substitution to synonymous substitution can reveal genes that evolve slowly from those that evolve quickly, thereby providing a quantitative measure on the selection force^15^,^61^. In the present study, we detected 120 positively selected genes through pair-wise comparisons of homologs among the three scale worms, which can be used for more detailed studies of genetic basis of adaptation to the deep-sea environment. These genes are involved in many fundamental biological processes, including cell structure and cell adhesion, indicating reshaping of cell structures to increase toughness might have contributed to the adaption to high hydrostatic pressure in the deep sea. Positive selection in genes related to energy-related process, chromatin structure and gene expression, protein metabolism and function may have allowed the deep-sea worms to adapt to the deep sea, since high hydrostatic pressure and low temperature can inhibit gene transcription and translation, and protein function^62^,^63^. Moreover, sulfide is a major toxic substance for deep-sea organisms^5^. Oxidation of sulfide is a way for deep-sea animals to avoid the damage by this chemical and this process is coupled with energy production directly in the mitochondria^64^. Nevertheless, only protein-coding genes were included in the positive selection analysis in this study, but positive selection could occur in the regulatory regions, such as promoters and enhancers. Further analysis of positive selection in scale worms can be done when genomes of these organisms become available.

Considering that gene expression is under precise regulation in living cells, and that several genes involved in chromatin structure and regulation of gene expression exhibit signals of positive selection, we also compared the highly expressed genes among the three species of scale worms. Even though the change in environment conditions during the sampling might have affected gene expression, especially the expression of heat-shock proteins, comparison of transcriptome data should reflect most of the intrinsic gene expression difference between the deep-sea and shallow-water species. Specifically, the higher expression of hemoglobins in the deep-sea species might be a response to the hypoxic condition in the chemosynthesis-based habitats, rather than a response to higher oxygen levels they experienced during ascending. Indeed, a previous study which also involved placing the samples in a partially insulated box, indicated that ascending to surface wouldn’t eliminate the difference in expression of hemoglobin genes for the polychaete *Ridgeia piscesae* collected from deep-sea habitats with different DO concentrations; they also found that levels of hemoglobin gene expression of animals processed immediately were not significantly different from those processed 10–12 h after^65^. Our study showed that, compared with the shallow-water species, a number of conserved gene families had remarkably different expression patterns between the deep-sea and shallow-water species. In particular, in both deep-sea species, hemoglobins, a group of oxygen-binding proteins, were much more abundant in gene number (>3 times) and had much higher expression levels (>10 times), likely an adaptation to the need of using oxygen more effectively and efficiently in the deep-sea chemosynthesis habitats that often experience hypoxia^1^,^36^. In addition, the dehydrogenase gene family, which participates in carbohydrate and lipid metabolism and ATP production, has the largest number of genes in *Lepidonotopodium* sp. and highest expression level in *B. pettiboneae*. This result is consistent with the high expression of dehydrogenases in deep-sea black scabbardfish^16^. Furthermore, several proteins that are involved in DNA repair and recombination (i.e. reverse transcriptase, DNA breaking-rejoining domains and integrase are involved in DNA repair and recombination) were only present in the top 10% expressed genes in the two deep-sea species. The two deep-sea species also had higher abundance of proteins with zinc finger and EF hand domains which are commonly present in protein-DNA binding proteins. Together with our finding that, chromatin structure and regulation of gene expression is the largest part of positively selected genes in the two deep-sea species, these results suggest that DNA repair, recombination and integration are more active in the two deep-sea species than in the shallow-water species. This is probably related to the DNA damage caused by various stressors from the extreme deep-sea environment^34^.

Gene gains and losses are assumed to play a crucial role in the adaptive evolution of animals^64^,^66^. Previous studies have indicated that gene duplication may have contributed to the adaptation to cold in marine fish: there were significantly more duplicate genes in the icefish *Chionodraco hamatus* compared with several model fish species^14^, and that many of the protein-coding genes that are specifically expressed in the Antarctic fish *Dissostichus mawsoni* corresponded to the up-regulated gene families when compared with non-Antarctic fish based on the transcriptome data^60^. Even though duplicated genes might be present in shallow-water species’ genome, transcriptomic data indicates that both deep-sea species have larger numbers of duplicated genes than the shallow-water species. Different pathways were enriched for putative duplicated genes in deep-sea scale worms, especially DNA metabolism process involved in DNA recombination, DNA integration and repair. For example, there were 18 duplicated integrases in *B. pettiboneae* and 32 in *Lepidonotopodium* sp., but only 3 in *H. imbricata*, further supporting that DNA recombination and repair activities are more active in the two deep-sea species, consistent with a previous study that showed frequent DNA strand breakage in animals collected from deep-sea hydrothermal vents^67^. Furthermore, the translation pathway was significantly enriched in deep-sea species for duplicated genes. Among them are 58 and 20 duplicated ribosomal proteins in *B. pettiboneae* and *Lepidonotopodium* sp. with a total of 123 and 56 genes, respectively, indicating more ribosomes might be needed for protein synthesis in the deep-sea species.

Hemoglobins have been a research focus in deep-sea scale worms because the involvement of gene duplication as a possible adaptive mechanism to hypoxia. As oxygen-binding proteins, hemoglobins are important for energy generation. Most deep-sea scale worms are red-blooded, indicating the presence of high concentrations of hemoglobins. It has been reported that tetra-domain hemoglobins from the deep sea have higher affinity for oxygen and can store oxygen for a longer period in complete anoxia when compared with single-domain hemoglobins from their littoral relatives^68^,^69^. Tetra-domain hemoglobins were previously known only in *B. seepensis* and *B. symmytilida*^23^. We found 22 hemoglobin sequences in the two deep-sea scale worms, including 17 in *B. pettiboneae* and 5 in *Lepidonotopodium* sp. Consistent with their importance in oxygen storage and transport, the hemoglobins of *B. pettiboneae* and *Lepidonotopodium* sp. accounted for higher percentages of highly expressed genes and had higher expression levels than the shallow-water species *H. imbricata. Branchipolynoe pettiboneae* also has a tetra-domain hemoglobin, and that each of its four domains is consistent in forming the sister clade of the corresponding domain of *B. seepensis* and *B. symmytilida*. This result indicates that the formation of the tetra-domain predated the divergence of the three species of *Branchipolynoe*, and that *B. seepensis* (a Gulf of Mexico species) and *B. symmytilida* (an eastern Pacific species) are more closely related to each other than to *B. pettiboneae* (a western Pacific species), indicating that the most recent common ancestor of *B. seepensis* and *B. symmytilida* spread from western Pacific and subsequently diverged to form these two species. More taxon sampling around the world would allow for testing this hypothesis about the geographical origin of tetra-domain hemoglobin. The phylogenetic analysis showed that the three *Branchipolynoe* species have both single domain and tetra-domain hemoglobins. The tetra-domain hemoglobins, having high affinity to oxygen^23^, were only present in *Branchipolynoe* species, consistent with previous findings. The result from the transcriptome that the tetra-domain hemoglobin is unique in *Branchipolynoe* spp. should be confirmed when full genome sequences from scale worms are available. Substitution analysis of hemoglobins in *Branchipolynoe* spp. by branch-site model using PAML showed that all of the single domain and tetra-domain hemoglobins except TD1 were positively selected, indicating divergent evolution of hemoglobins has contributed to the adaptation in deep-sea scale worms. Specifically, the amino acid 66 C (Cysteine) in the hemoglobin is under positive selection, the same with some extracellular globins from other annelids living in sulfide-rich environments^70^. Cysteine in the same position was proposed to be involved in the reversible binding of sulfide in the deep-sea tube worm *Riftia*^71^. Interestingly, although *Lepidonotopodium* sp. does not possess tetra-domain hemoglobin, the expression level of the other type of hemoglobin was almost four times that of *B. pettiboneae*, indicating this scale worm has adopted a different strategy to efficiently obtain oxygen in the hypoxic deep-sea environment.

## Conclusions

The first two deep-sea scale worm transcriptomes were reported in this paper. A total of 120 positively selected genes were found in pair-wise comparisons among the three species. They are involved in different fundamental biological processes, including cell structure and cell adhesion, energy-related process, chromatin structure and gene expression, signal pathway, protein metabolism and function, and transport. Among the top 10% expressed genes, globin genes in the deep-sea species were more abundant and highly expressed than in the shallow-water species; genes related to DNA repair, recombination and integration were only found in the deep-sea species. For putatively duplicated genes, GO terms related to DNA recombination and metabolism, and gene expression were only enriched in the deep-sea species. The phylogenetic analysis showed that the tetra-domain hemoglobin is unique to *Branchipolynoe*, and evolutionary analysis showed that three of the tetra-domains had a clear signal of positive selection. Although *Lepidonotopodium* sp. does not possess tetra-domain hemoglobin, its expression level of single-domain hemoglobin was four times as high as that in *B. pettiboneae*. The two deep-sea scale worms therefore have evolved alternative strategies in adapting to the hypoxic deep-sea environment. Taken together, our analyses revealed a number of genomic differences between the deep-sea and shallow-water scale worms. More similar comparative studies will enhance our understanding of the genomic basis of adaptation to the extreme environments in deep-sea chemosynthetic ecosystems.

## Additional Information

**How to cite this article:** Zhang, Y. *et al*. Adaptation and evolution of deep-sea scale worms (Annelida: Polynoidae): insights from transcriptome comparison with a shallow-water species. *Sci. Rep.* **7**, 46205; doi: 10.1038/srep46205 (2017).

**Publisher's note:** Springer Nature remains neutral with regard to jurisdictional claims in published maps and institutional affiliations.

## Supplementary Material

Supplementary Information

Supplementary Tables

## Figures and Tables

**Table 1:** Raw data and assembly information in the transcriptomes of three scale worms.

Parameters	*B. pettiboneae*	*Lepidonotopodium sp.*	*H. imbricata*
Total bases	3.8 G	7.0 G	3.9 G
Read length	100 bp	150 bp	100 bp
Number of reads	38,159,052	46,701,686	39,257,588
Number of clean reads	37,548,398 (98.4%)	39,579,324 (84.7%)	35,823,606 (91.3%)
Number of contigs^#^	153,339	225,709	98,806
Shortest contig	224	224	224
Longest contig	11,622	18,505	18,407
Average contig length	521.49	738.60	536.55
N50 value	583	1,198	605
Predicted genes	38,734	79,930	36,001
Annotated genes*	14,998 (38.7%)	32,915 (41.2%)	17,493 (48.6%)
Proteins with GO annotation	8,386	20,849	10,343

^#^Contigs: sequences in Trinity.fasta, the output file of Trinity. *Annotated genes: predicted proteins in the output file of InterProScan.

**Table 2:** The proportions (%) of amino acid of transcriptomes from *B. pettiboneae* (P_B_), *Lepidonotopodium* sp (P_L_) and *H. imbricata* (P_H_).

	AA	P_B_	P_L_	P_H_	P_B_-P_L_	P_B_-P_H_	P_L_-P_H_
Non polar AA	Ala	7.080902	7.023033	6.887126	0.05787	0.193776	0.135907
	Val	6.757875	6.700042	6.696981	0.05783	0.060894	0.003061
	Ile	4.693721	4.50876	4.412114	0.18496	0.281607	0.096647
	Leu^**#**^	9.189067	9.211126	8.645725	−0.0221	0.543342	0.565401
	Met	2.539144	2.593958	2.529529	−0.0548	0.009615	0.064429
	Phe	3.608689	3.489103	3.416445	0.11959	0.192244	0.072658
	Trp	1.344377	1.313756	1.210222	0.03062	0.134155	0.103534
Special AA	Gly^#^	6.275816	6.356668	6.913042	−0.0809	−0.63723	−0.55637
	Pro	5.756952	5.927207	5.851982	−0.1703	−0.09503	0.075226
	Cys	2.737011	2.473187	2.254504	0.26382	0.482508	0.218683
Negative charged AA	Asp^#^	4.790209	4.890573	5.322978	−0.1004	−0.53277	−0.43241
	Glu^#^	5.060994	5.400542	6.09626	−0.3395	−1.03527	−0.69572
Positive charged AA	His^#^	3.196353	3.067233	2.648607	0.12912	0.547746	0.418626
	Lys	4.995021	4.80515	5.311784	0.18987	−0.31676	−0.50663
	Arg^#^	6.461761	6.416667	5.970063	0.04509	0.491698	0.446604
Polar uncharged AA	Thr	6.353314	6.326381	6.43108	0.02693	−0.07777	−0.1047
	Tyr	2.613485	2.52377	2.540749	0.08972	0.072736	−0.01698
	Gln	4.026075	4.11024	4.236499	−0.0842	−0.21042	−0.12626
	Asn	3.648973	3.624642	3.694103	0.02433	−0.04513	−0.06946
	Ser	8.530669	8.932094	8.682798	−0.4014	−0.15213	0.249297

AA: amino acid.

^**#**^The differences are larger than 0.4% between deep-sea and shallow-water species.

**Table 3:** Summary of the orthologous genes and Ka/Ks analyses in pair-wise comparison among three scale worms.

	Bran vs Harm	Lepi vs Harm	Bran vs Lepi
No. of orthologs	5,494	6,851	7,311
No. of orthologs after filtering	1,161	1,801	3,269
mean Ka value	0.208	0.201	0.151
mean Ks value	0.593	0.586	0.672
mean Ks/Ks value	0.659	0.637	0.545
No. of orthologs with Ka/Ks > 1	192	306	416
mean Ka value	0.500	0.421	0.360
mean Ks value	0.266	0.222	0.170
mean Ka/Ks value	2.704	2.475	3.141
No. of orthologs Ka/Ks > 1 (with annotation)	51	40	29
mean Ka value	0.642	0.565	0.579
mean Ks value	0.370	0.337	0.319
mean Ka/Ks value	2.017	1.889	2.135

Bran: *B. pettiboneae*; Harm: *H. imbricata*; Lepi: *Lepidonotopodium* sp.

**Table 4:** Positive selection analysis of single-domain and tetra-domain hemoglobins in *Branchipolynoe* spp.

	TD1-4	TD1	TD2	TD3	TD4
**InL**	−2631.577	−2641.405	−2637.472	−2644.831	−2637.171
**κ**	1.734	1.713	1.691	1.755	1.726
**np**	32	32	32	32	32
ω_0_	0.219 (65.8%)	0.232 (78.9%)	0.230 (75.9%)	0.248 (76.96%)	0.231 (76.3%)
ω_1_	1 (16.0%)	1 (21.1%)	1 (15.4%)	1 (19.3%)	1 (17.4%)
ω_2a_	40.993 (14.6%)	1 (0)	10.305 (3%)	76.832 (3%)	∞ (5.1%)
ω_2b_	40.993 (3.5%)	1 (0)	10.305 (0.7%)	76.832 (0.7%)	∞ (1.2%)
**2ΔlnL**^**#**^	12.439***	0^NS^	4.820*	7.148**	7.415**
**Selected sites**	31A; 66C; 112 L				11S

InL: integral nonlinearity, the maximum deviation between the ideal output and the actual output level; κ: transition/transversion ratio; np: number of parameters estimated by the model; ω_0_: estimated ω for the category of sites under purifying selection; ω_1_: estimated ω for sites under neutral evolution; ω_2a_: estimated ω for sites under positive selection in the foreground branches against background branches under purifying selection; ω_2b_: estimated ω for sites under positive selection in the foreground branches against background branches under neutral evolution.

^#^*P *<* *0.05: *P < 0.01: **P < 0.001. NS: not significant.
