# Supplementary material for: Adaptation and evolution of deep-sea scale worms (Annelida: Polynoidae): insights from transcriptome comparison with a shallow-water species
Source: Sci Rep. 2017 Apr 11;7:46205. doi: 10.1038/srep46205 (PMC5387418; doi:10.1038/srep46205)
Supplement: Supplementary Information [file srep46205-s1.pdf]

## Additional information

### Adaptation and evolution of deep-sea scale worms (Annelida: Polynoidae): insights from transcriptome comparison with a shallow-water species

Yanjie Zhang[1], Jin Sun[2], Chong Chen[3], Hiromi K. Watanabe[4], Dong Feng[5], Yu Zhang[6], Jill M.Y. Chiu[1], Pei-Yuan Qian[2,*], Jian-Wen Qiu[1,*]

[1] Department of Biology, Hong Kong Baptist University, Hong Kong, P. R. China

[2] Division of Life Sciences, The Hong Kong University of Science and Technology, Clear Water Bay, Hong Kong, P. R. China

[3] Department of Subsurface Geobiological Analysis and Research, Japan Agency for Marine-Earth Science and Technology, 2-15 Natsushima-cho, Yokosuka, Kanagawa, 237-0061, Japan

[4] Department of Marine Biodiversity Research, Japan Agency for Marine-Earth Science and Technology, 2-15 Natsushima-cho, Yokosuka, Kanagawa, 237-0061, Japan

[5] CAS Key Laboratory of Marginal Sea Geology, South China Sea Institute of Oceanology, Chinese Academy of Sciences, Guangzhou 510301, P. R. China

[6] College of Bio and Marine Sciences, Shenzhen University, Shenzhen, P. R. China

*Corresponding authors: Jian-Wen Qiu (qiujw@hkbu.edu.hk), Pei-Yuan Qian (boqianpy@ust.hk)

**Supplementary information, PickUpLong.py**

Note: This script is used to pick up the longest amino acid sequence from the translated DNA sequences generated from Transdecoder. This script was written by Dr. Jin Sun.

```
###############################################################################
import os
import math


sSourceFileName = "TaaGetorf.TXT";
sTargetFileName =    sSourceFileName.split(".")[0]\
        + "_target."\
        + sSourceFileName.split(".")[1];

fSourceFile = file(sSourceFileName,"r");
fTargetFile = file(sTargetFileName,"w");


def writeFilteredDataList(_lFilteredDataList,_fTargetFile):
    nListLen = len(_lFilteredDataList);
    if nListLen<=0:
        return;
    print;

    for i in range(1,min(nListLen,4)):
        _fTargetFile.write(">"+lFilteredDataList[0] + "_" + _lFilteredDataList[i][0]+"\n");
        _fTargetFile.write(_lFilteredDataList[i][1]+"\n");
        print "      "+str(i)+":"+">"+lFilteredDataList[0] + "_" + _lFilteredDataList[i][0] +":"+
str(len(_lFilteredDataList[i][1]));

lFilteredDataList = [];
bIsSameType = False;
sDNATitle = "";

for sLine in fSourceFile:
    sNowDNATag = "";

    sLine = sLine.strip();
    if sLine.find(">")!=-1:
        sLine = sLine[1:];
        sNowDNATag = sLine.split("_")[0];
        sDNATitle = sLine.split("_")[1];
```

```
            if len(lFilteredDataList)>0 and cmp(lFilteredDataList[0],sNowDNATag)==0:
                bIsSameType = True;
            else:
                bIsSameType = False;

            if bIsSameType==False:
                nListLen = len(lFilteredDataList);
                if nListLen>0:
                    writeFilteredDataList(lFilteredDataList,fTargetFile);
                    lFilteredDataList = [];
                lFilteredDataList.append(sNowDNATag);

        else:
            lListData = [];
            lListData.append(sDNATitle);
            lListData.append(sLine);

            nListLen = len(lFilteredDataList);
            if nListLen<0:
                print;
            elif nListLen==1:
                lFilteredDataList.append(lListData);
            else:
                nLineLen = len(sLine);
                for i in range(1,nListLen):
                    nLenOfLineFormList = len(lFilteredDataList[i][1]);
                    if nLineLen>nLenOfLineFormList:
                        lFilteredDataList.insert(i,lListData);
                        break;
                if len(lFilteredDataList)==nListLen:
                    lFilteredDataList.append(lListData);

    writeFilteredDataList(lFilteredDataList,fTargetFile);


    fTargetFile.close();
    fSourceFile.close();

    ######################################################################################
```

**Supplementary Fig. S1.**

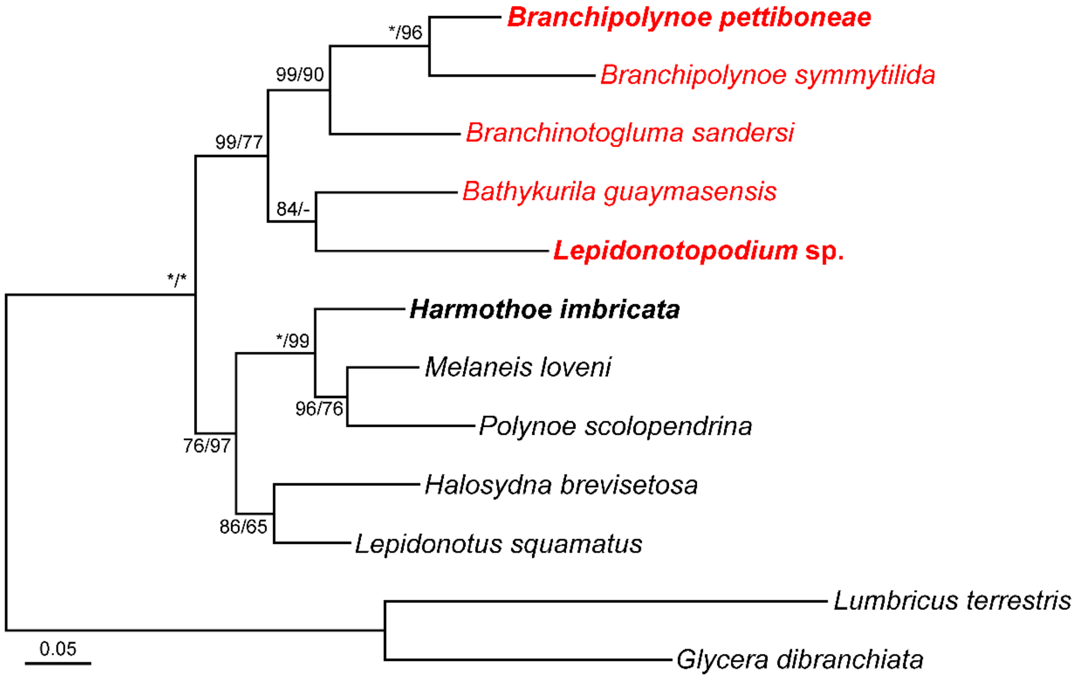


**Supplementary Fig. S1.** Phylogenetic tree of several polynoids based on several genes (i.e. COI and 16S, 18S, 28S rRNA). It was constructed following the method described in Phylogenetic analysis in the section of Materials and methods. GTR+I+G model was used in ML tree. Three species (bold) compared in this paper were included in the phylogenetic tree. Sequences from *Lumbricus terrestris* and *Glycera dibranchiata* served as outgroups. Numbers near branches represent MP/ML bootstrap values based on 1,000 iterations, with 100 (indicated using an asterisk) as the highest value. Bootstrap values below 50 are shown only as a short dash due to the weak support. Deep-sea species are highlighted in red color. Accession numbers of related sequences (COI, 16S, 18S, 28S) are shown as follows: *Lumbricus terrestris*: HQ024638, KM987009, AJ272183, HQ691218; *Glycera dibranchiata*: HQ024038, GQ478120, AY995208, AY995207; *Harmothoe imbricata*: GQ478931, AY340463, AY340434, AY340400; *Lepidonotus squamatus*: AY894316, JN852903, AY176290, JN852865; *Halosydna brevisetosa*: AY894313, JN852895, JN852827, JN852857; *Melaneis loveni*: JN852936, JN852905, JN852835, JN852867; *Polynoe scolopendrina*: JN852940, JN852909, JN852839, JN852870; *Branchinotogluma sandersi*: JN852923, JN852889, JN852821, JN852851; *Branchipolynoe symmytilida*: AY646057, AF315055, -, - ; *Bathykurila guaymasensis*: DQ074766, -, DQ074765, -. The sequences of *Branchipolynoe pettiboneae* and *Lepidonotopodium* sp. were obtained from assembled transcriptome by BLAST.
